# Dual induction of apoptotic and autophagic cell death by targeting survivin in head neck squamous cell carcinoma

**DOI:** 10.1038/cddis.2015.139

**Published:** 2015-05-28

**Authors:** L Zhang, W Zhang, Y-F Wang, B Liu, W-F Zhang, Y-F Zhao, A B Kulkarni, Z-J Sun

**Affiliations:** 1 The State Key Laboratory Breeding Base of Basic Science of Stomatology & Key Laboratory of Oral Biomedicine Ministry of Education, School and Hospital of Stomatology, Wuhan University, Wuhan, China; 2 Department of Oral and Maxillofacial-Head and Neck Oncology, School and Hospital of Stomatology, Wuhan University, Wuhan, China; 3 Functional Genomics Section, Laboratory of Cell and Developmental Biology, National Institute of Dental and Craniofacial Research, National Institutes of Health, Bethesda, MD, USA

## Abstract

Survivin is ubiquitously expressed in patients with head neck squamous cell carcinoma (HNSCC) and is associated with poor survival and chemotherapy resistance. Sepantronium bromide (YM155) is a selective survivin suppressant that exhibits potent antitumor activities by inducing apoptosis and autophagy in various types of cancer. However, the curative effects and underlying mechanisms of YM155 in HNSCC remain unclear. This study showed that survivin overexpression positively correlated with p-S6, p-Rb and LAMP2 but negatively correlated with the autophagic marker LC3 in human HNSCC tissues. *In vitro* studies revealed that YM155 triggered apoptosis of HNSCC cells in mitochondria and death receptor-dependent manner. The treatment also significantly enhanced autophagy by upregulating Beclin1, which led to cell death. YM155 not only downregulated the expression of survivin but also remarkably suppressed the activation of the mTOR signaling pathway *in vitro* and *in vivo*. YM155 displayed potent antitumor activities in both CAL27 xenograft and transgenic HNSCC mice models by delaying tumor onset and suppressing tumor growth. Furthermore, YM155 combined with docetaxel promoted tumor regression better than either treatment alone without causing considerable body weight loss in the HNSCC xenograft models. Overall, targeting survivin by YM155 can benefit HNSCC therapy by increasing apoptotic and autophagic cell death, and suppressing prosurvival pathways.

Head and neck squamous cell carcinoma (HNSCC), which occurs in the oral cavity, oropharynx, larynx and hypopharynx, is the sixth most common malignancy worldwide.^1^ It affects 600 000 new patients each year, which accounts for over 90% of head and neck cancers.^2, 3^ The current preferred therapy for HNSCC is combined surgery, radiotherapy, chemotherapy and biotherapy; however, the 5-year survival rate is still <50%, and the long-term survival rate has only marginally improved.^4, 5, 6^ As an important hallmark of head and neck cancer, apoptosis resistance restricts the efficacy of traditional therapies.^7^ *Survivin* (also called *BIRC5*) inhibits apoptosis-related proteins, regulates cell division, relates to stress response and promotes tumor-associated angiogenesis in HNSCC.^8^ Survivin is also associated with high-grade and advanced HNSCC, poor survival, high recurrence rate and chemotherapy and radiation resistance. Therefore, targeting survivin is promisingly beneficial for head and neck cancer therapies.

Sepantronium bromide (YM155) is a small imidazolium-based compound (1-(2-methoxyethyl)-2-methyl-4,9-dioxo-3-(pyrazin-2-ylmethyl)-4,9-dihydro-1H-naphtho[2,3-d]imidazolium bromide) that selectively suppresses survivin expression^9^ and displays potent anticancer activities against various types of cancer.^10, 11, 12^ Previous researches have focused on that YM155 induced the apoptosis by downregulating survivin in cancer cells.^10, 13, 14^ Recent studies including ours have demonstrated YM155 also triggered autophagy in cancer cells.^15, 16, 17^ Macroautophagy or autophagy is considered to be another type of programmed cell death wherein proteins are degraded by autophagosomes and lysosomes.^18^ Autophagy also has an important role in tumorigenesis.^19^ Autophagy shares several regulatory systems and common pathways with apoptosis; thus, autophagy is closely linked with apoptosis. *Beclin1* (*ATG6*), an autophagy-specific gene that is essential for autophagosome induction and elongation, interacts with several apoptosis-related genes, such as *bcl-2*, *bcl-xl* and survivin.^20^ Therefore, YM155 may not only induce the apoptosis but also affect the autophagy in HNSCC.

The present study investigated the antitumor effects of YM155 on HNSCC *in vitro* and *in vivo* through dual induction of apoptotic and autophagic cell death. Although it specifically suppressed the expression of survivin, we here proved YM155 also targeted the mTOR signaling pathway, which was the principal regulator of cancer cell survival and autophagy. Most importantly, in an inducible tissue-specific spontaneous HNSCC mouse model with 100% penetrance by the combined deletion of *Tgfbr1* and *Pten* (*Tgfbr1/Pten* 2cKO) in the oral mucosa^21^ with ubiquitous activation of the Akt/mTOR/survivin pathway,^22^ YM155 exerted significant therapeutic effects by delaying tumor onset and suppressing tumor growth. This finding coincided with the xenograft results. Finally, the effects of YM155 when combined with traditional chemotherapeutic agent were also determined.

## Results

### YM155 induces both apoptotic and non-apoptotic cell death in HNSCC

**Figure 1:**
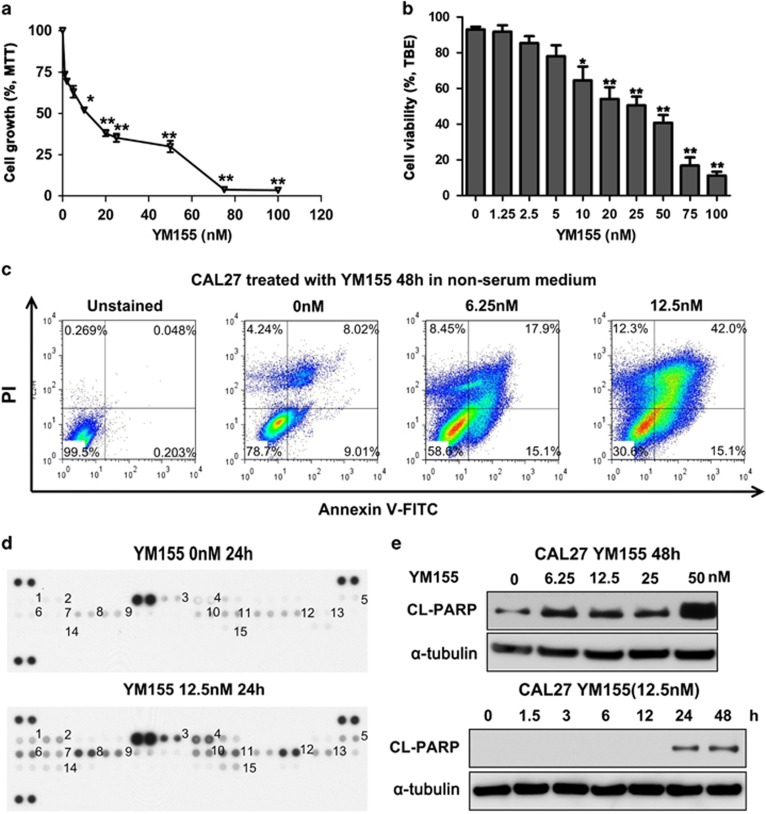
YM155 induces apoptoic cell death in HNSCC cell line. (**a**) IC50 of YM155 in CAL27 cells; *P< 0.05, **P< 0.01 (**b**) TBE assays revealed the cell death induced by YM155; *P< 0.05, **P< 0.01. (**c**) Representative dot plot of flow cytometry showed YM155 enhanced both apoptotic and non-apoptotic cell death in CAL27 cells. (**d**) Human apoptosis antibody array showed the increase of both mitochondria and cell death receptor-dependent apoptosis by 12.5 nM Ym155 treatment in CAL27 cells. (**e**) Western blot analysis cleaved-PARP (CL-PARP) expression in CAL27 cells after YM155 treatment

YM155 is the widely used suppressant for survivin inhibition. To examine the possible antiproliferative role of survivin inhibition in HNSCC, we first determined the expression of survivin and related kinases in human HNSCC cell lines. As shown in Supplementary Figure 1a, HNSCC cell lines exhibited upregulated expression of survivin and increased phosphorylation of p-Rb^S780^ and p-S6^S235/236^ as compared with human oral keratinocytes (HOK). We then determined the IC50 values of the survivin inhibitor YM155 in HNSCC cell lines. As shown in Figure 1a and Supplementary Figure 1b, the IC50 values of YM155 for the CAL27 and HSC3 cell lines were 12.7 and 19.1 nM, respectively. The cell viability was estimated by trypan blue exclusion (TBE) assays, suggesting at the concentration of 10 nM, YM155 caused signficant cell death. Then, the suppression of survivin was measured in both protein and mRNA levels (Supplementary Figure 1c). Annexin V-FITC/PI double staining was then performed to measure apoptosis of CAL27 cells after YM155 treatment. The population of Annexin V+/PI+ late-apoptotic cells significantly increased after treatment with 6.25 nM YM155 for 24 h. The increase in the population of Annexin V−/PI+ necrotic cells indicated that a high YM155 dose might exert potential cytotoxicity against HNSCC (Figure 1c). To confirm the apoptotic effect of YM155 on HNSCC, we utilized a high-throughput antibody array with apoptotic and anti-apoptotic factors and examined their expressions in CAL27 cells treated with YM155 as compared with the PBS control. The levels of the apoptotic factors including bad, bax, cleaved caspase, cytochrome C, TRAIL R1/R2 and FADD were increased in the YM155-treated HNSCC cells (Figure 1d and quantification in Supplementary Figure 1d). To validate the antibody array data, we performed ELISA and confirmed that YM155 increased cytochrome C release (Supplementary Figure 1e) and caspase-9 activities (Supplementary Figure 1f) in both CAL27 and HSC3 cells. In addition, YM155 increased cleavage of poly(ADP-ribose) polymerase (PARP) in CAL27 cells (Figure 1e). These results confirmed that survivin inhibition by YM155 promoted the apoptotic cell death of HNSCC cell lines *in vitro*.

### YM155 induces autophagy in human HNSCC

**Figure 2:**
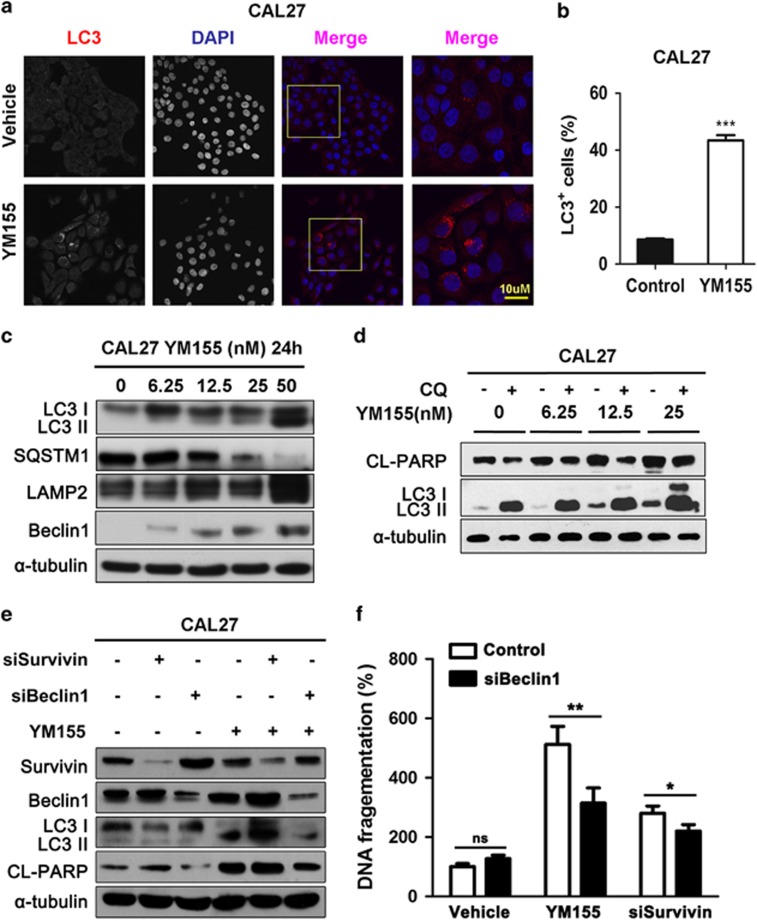
YM155 induces autophagy in human HNSCC cell line. (**a**) Representative photographs of LC3 dot punctuate indicated YM155 enhanced autophagy in CAL27 cell line after 6 h YM155 treatment. Scale bar, 10 *μ*m. (**b**) Quantitative analysis of the ratio of LC3+ cells among total cells; ***P< 0.001. (**c**) Western blot analysis indicated the increase of LC3II, LAMP2, Beclin1 protein but decrease of SQSTM1 expression in CAL27 cells by YM155 treatment. (**d**) Western blot showed inhibiting the fusion of autophagosome and lysosome by CQ suppressed the apoptosis indicated by the decreased CL-PARP expression in CAL27 cell line with YM155 or vehicle treatment. (**e**) Western blot showed RNA interference against Beclin1 suppressed the YM155 or survivin siRNA increased LC3II/I ratio and decreased CL-PARP expression. (**f**) DNA fragmentations of CAL27 cells were measured to evaluate the contribution of autophagy in YM155-induced cell death; *P< 0.05, **P< 0.01

Recent studies have shown that autophagy might be another type of programmed cell death.^23^ To further explore the role of survivin inhibition in HNSCC autophagy, we measured LC3 dot punctate (Figures 2a and b), a gold standard in detecting autophagy, in CAL27 cells in response to YM155 treatment. The increased LC3II/I ratio, Beclin1 and LAMP2 expression, and reduced expression of SQSTM1, a 62 KD protein degraded only by autophagy, were confirmed in both CAL27 (Figure 2c) and HSC3 cells (Supplementary Figure 2a). The results also indicated that YM155 increased the autophagic flux (Supplementary Figure 2b). The inhibition by YM155 might increase LAMP2 at the early stage (e.g., 1.5 h after YM155) but then decrease LAMP2 gradually. In order to further confirm the biological function of YM155-induced autophagy in HNSCC, CQ was used as a putative autophagy inhibitor to block the atuophgic flux. As shown in Figure 2d, autophagy led HNSCC cells to death, as indicated by a decrease in the cleaved PARP when combined treatment with CQ in HNSCC. Moreover, RNA interference targeted on Beclin1 comfirmed YM155 (12.5 nM)-induced autophagy was dependent on Beclin1 expression (Figure 2e). Considering that the direct binding between Beclin1 and survivin can regulate apoptosis,^24^ we explored whether or not this interaction contributed to YM155-induced autophagy. As shown, the suppression of survivin caused the release of Beclin1, which might serve as an additional mechanism to enhance autophagy (Supplementary Figure 3a). Further, the cytotoxic effect of YM155 in CAL27 after suppression of autophagy were explored. As shown in Figure 2f and Supplementary Figure 3b, siRNA against Beclin1 only slightly reduced the cell death caused by siRNA against survivin, but significantly decreased the cell death induced by YM155 (12.5 nM), suggesting that YM155 caused cytotoxicity was partially dependent on autophagy. Therefore, we concluded that YM155-induced cell death in both survivin and autophagy-dependent manner.

### YM155 reduces the phosphorylation of mTOR in human HNSCC

**Figure 3:**
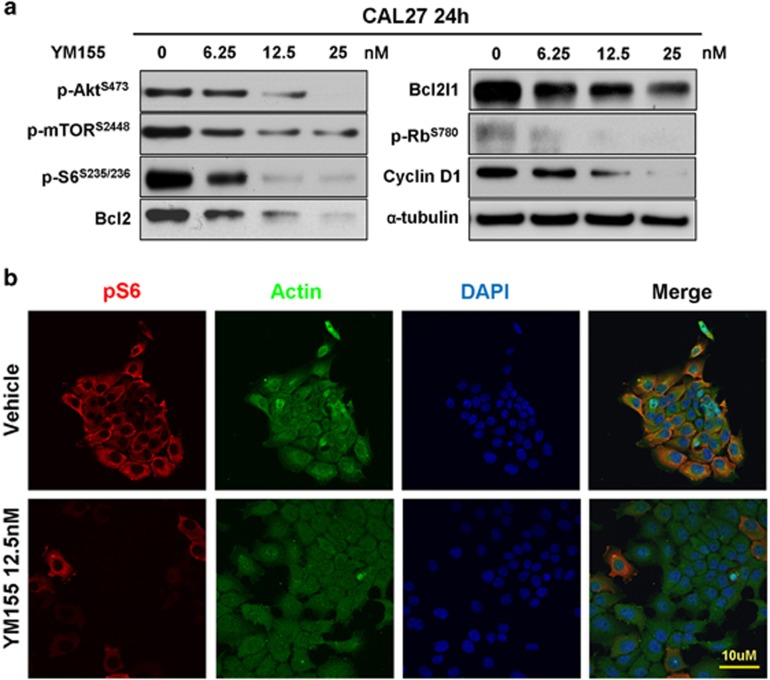
YM155 decreases phosphorylation of mTOR in HNSCC cell line. (**a**) CAL27 cells were treated with indicated doses YM155 for 24 h. Western blot analysis showed the weakened phosphorylation of p-Akt, p-mTOR, p-S6 accompanied with decreased cell cycle markers including p-Rb, cyclin D1 and prosurvival markers Bcl2 and Bcl2l1. (**b**) Immunofluorescence showed the decreased cytoplasm and membrane expression of p-S6 in YM155 group

The phosphorylation of Akt/mTOR is a widespread molecular event, and mTOR is a master molecule for autophagy regulation. We observed the reduced phosphorylation of p-Akt, p-mTOR and p-S6 in the YM155-treated CAL27 cells (Figure 3a). Meanwhile, the downstream targets and anti-apoptotic factors Bcl2 and Bcl2l1 (Figure 3a) decreased in the YM155-treated cells. Similarly, the phosphorylation of Rb decreased with decreasing cyclin D1. This observation was also confirmed through the immunofluorescence staining of p-S6 in the YM155-treated cells (Figure 3b).

### Targeting survivin by YM155 suppresses the progression of HNSCC in xenograft and transgenic mice models

**Figure 4:**
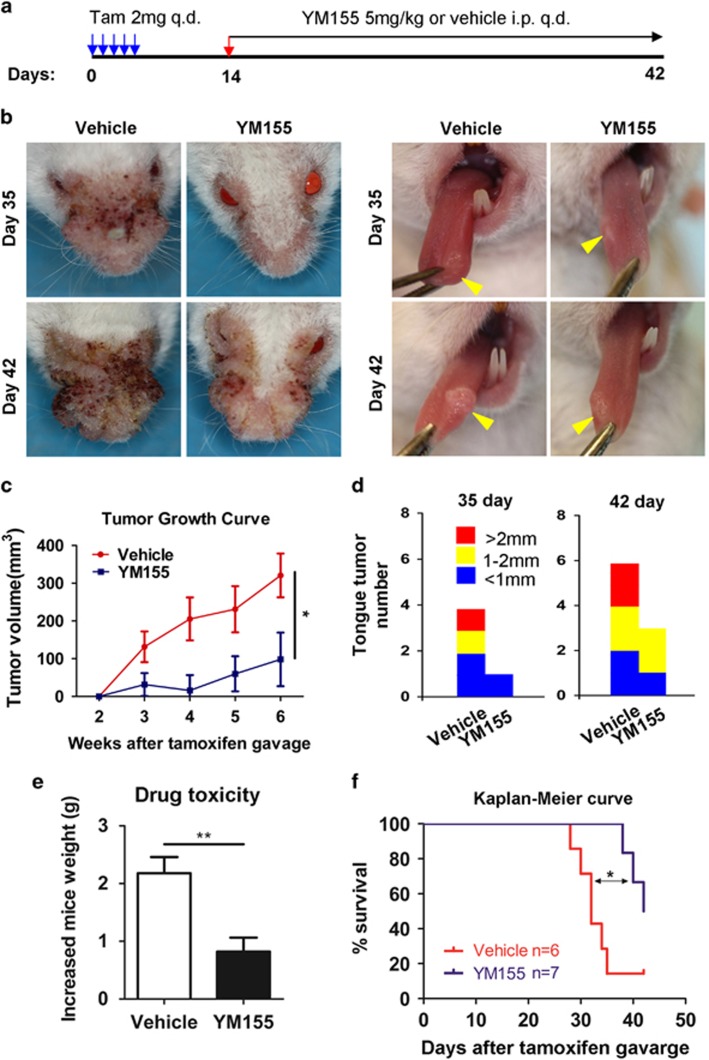
Targeting survivin by YM155 delays onset of HNSCC in *Tgfbr1/Pten* 2cKO mice. (**a**) *Tgfbr1/Pten* 2cKO mice bearing carcinoma were treated with 5 mg/kg YM155 intraperitoneally (i.p) daily for 14 days or vehicle control treated. (**b**) Representative photos of mice tumor with external head and neck (left) and tongue (right) after treatment with YM155 or vehicle in day 35 and day 42 after tumor. (**c**) Tumor growth measured every other day. Mean±S.E.M. of tumor sizes were shown; *P< 0.05. (**d**) Tongue tumor number were assessed in YM155 and control group. (**e**) Drug toxicity were assessed by gained body weight of *Tgfbr1/Pten* 2cKO mice in each group; Mean±S.E.M., **P<0.01. (**f**) Kaplan–Meier curve of survival of *Tgfbr1/Pten* 2cKO mice; Mean±S.E.M.; *P<0.05

To determine whether or not targeting survivin in HNSCC tumorigenesis can block or delay the occurrence of HNSCC, we performed a chemopreventive study on *Tgfbr1/Pten* 2cKO mice with a 100% rate of developing spontaneous HNSCC after 4 weeks of induction with considerably high survivin expression.^25^ The induction of HNSCC tumor onset in *Tgfbr1/Pten* 2cKO mice has been previously described.^25^ Figure 4a shows the induction and drug administration strategies. At 2 weeks after the last tamoxifen oral gavage, the mice were treated with YM155 (5 mg/kg intraperitoneal injection twice per week) or vehicle for 2 weeks. YM155 significantly (*P*<0.05, *n*=7) prevented the tumorigenesis (Figure 4b and c) and reduced the tumor burden (Figure 4d) of *Tgfbr1/Pten* 2cKO mice compared with the vehicle-only group (*n*=6). No significant weight loss was observed in the immunosufficient mice, suggesting that YM155 exerted no potential toxicity (Figure 4e). Moreover, no animal died in the treatment group during the experiment. Hence, YM155 benefited the survival of the mice at a tolerable dose (Figure 4f). Overall, these data indicated that the increase of survivin expression is an early event in HNSCC tumorigenesis and that the blockade of survivin by YM155 can decelerate the HNSCC onset in immunosufficient mice.

To further confirm the role of survivin in HNSCC tumorigenesis, we also established a mouse model of heterotopic xenograft tumor derived from CAL27 cells in response to YM155. The mice received treatment at day 7 postimplantation in the xenograft model (Supplementary Figure 4a). As shown in Supplementary Figures 4b and c, the YM155-treated group showed significantly inhibited tumor compared with the vehicle-only group until day 28. The indicated YM155 dose exerted no significant toxicity to the mice (data not shown). YM155 treatment significantly delayed the tumorigenesis of the CAL27 xenograft (Supplementary Figure 4d) and reduced the tumor burden (Supplementary Figure 4e). Therefore, blocking survivin can effectively prevent tumor growth.

More, the synergistic efficacy of YM155 in combination with 20 nM docetaxel was examined in CAL27 human HNSCC xenografts. As shown in Supplementary Figures 5a and b, docetaxel treatment increased the expression of survivin *in vitro*, which might contribute to the chemoresistance of HNSCC. Then, the synergistic efficacy of YM155 in combination with 20 nM docetaxel was examined in CAL27 human HNSCC xenografts. YM155 was administered at 5 mg/kg/d with 14 d of infusion to recapitulate the clinical dosing and steady-state serum concentrations in humans. YM155 combined with docetaxel significantly inhibited tumor growth in CAL27-established tumors compared with either drug alone (*P*<0.0001). Each monotherapy treatment induced tumor regression, followed by successive tumor regrowth during the observation period (Supplementary Figures 5c). No significant decrease in body weight was observed in the combination group as compared with the docetaxel group (Supplementary Figure 5d). These results indicated that YM155 in combination with docetaxel was tolerated by mice and enhanced the tumor response to docetaxel *in vivo*.

### YM155 inhibits the prosurvival pathway Akt/mTOR *in vivo*

**Figure 5:**
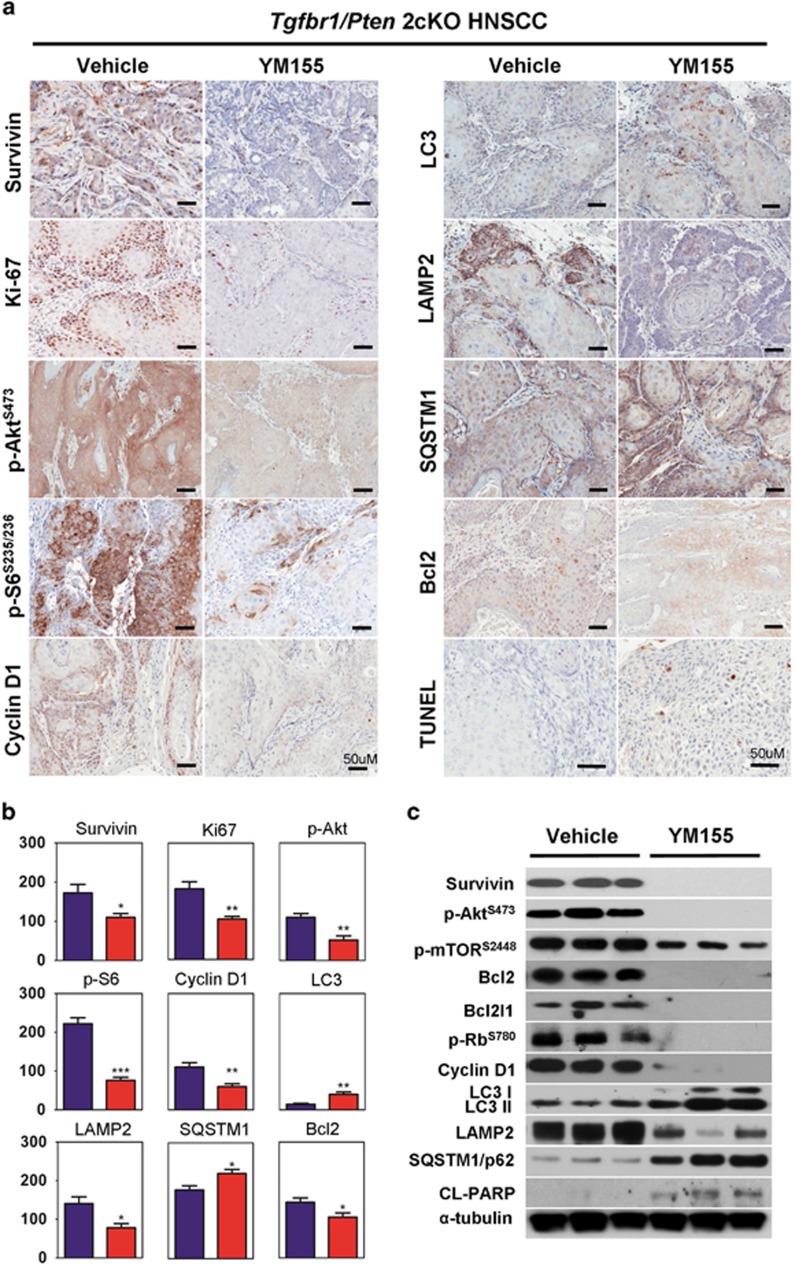
YM155 inhibits prosurvival pathway in *Tgfbr1/Pten* 2cKO mice. (**a**) Histoimmunochemical staining of survivin, Ki-67, p-Akt, p-S6, Cyclin D1, LC3, LAMP2, SQSTM1, Bcl2, TUNEL. *n*=5; Scale bar, 50 *μ*m. (**b**) Quantitative of histoscore of Survivin, Ki67, p-Akt, p-S6, Cycline D1, LC3, LAMP2, SQSTM1, Bcl2 expression in vehicle group and YM155-treated group from *Tgfbr1/Pten* 2cKO mice; Mean±S.E.M.; *P<0.05, **P<0.01, ***P<0.001, *n*=5 from each group. (**c**) Protein was extracted and interrogated by western blots for survivin, p-Akt, p-mTOR, Bcl2, Bcl2l1, p-Rb, Cyclin D1, LC3I, LC3II, LAMP2,SQSTM1 and CL-PARP as compared with the vehicle treated group, GADPH were used as a loading control

Immunohistochemical analysis was further performed to explore the molecule events in 2cKO mice after YM155 treatment. The results in Figure 5a and quantities in Figure 5b show that Ki67 and cyclin D1 were significantly downregulated in the YM155-treated group as compared with the vehicle-only group, strongly evidencing proliferation restraint of YM155. The expression levels of p-Akt and p-S6 were then evaluated in tumor tissues from YM155-treated 2cKO mice. Compared with the vehicle-only group, the YM155-treated group displayed significantly lower expression levels of p-Akt and p-S6. Moreover, western blot analysis confirmed the inhibition of the expression of survivin and phosphorylation of p-Akt and p-S6 at the protein level (Figure 5c). YM155 treatment increased LC3 but reduced LAMP2. This result demonstrated the accumulation of autophagosome, which was further proved by detecting SQSTM1 expression using western blot analysis (Figure 5c). Moreover, treatment with 5-nM YM155 reduced expression of Bcl2, which could inhibit autophagy by binding to Beclin1. These findings indicated that autophagy was increased (Figures 5a and c). Therefore, YM155 may increase autophagy through Akt/mTOR inhibition and Bcl2 suppression.

### Increased survivin level correlates with p-S6, p-Rb, LAMP2 and LC3 in human HNSCC

**Figure 6:**
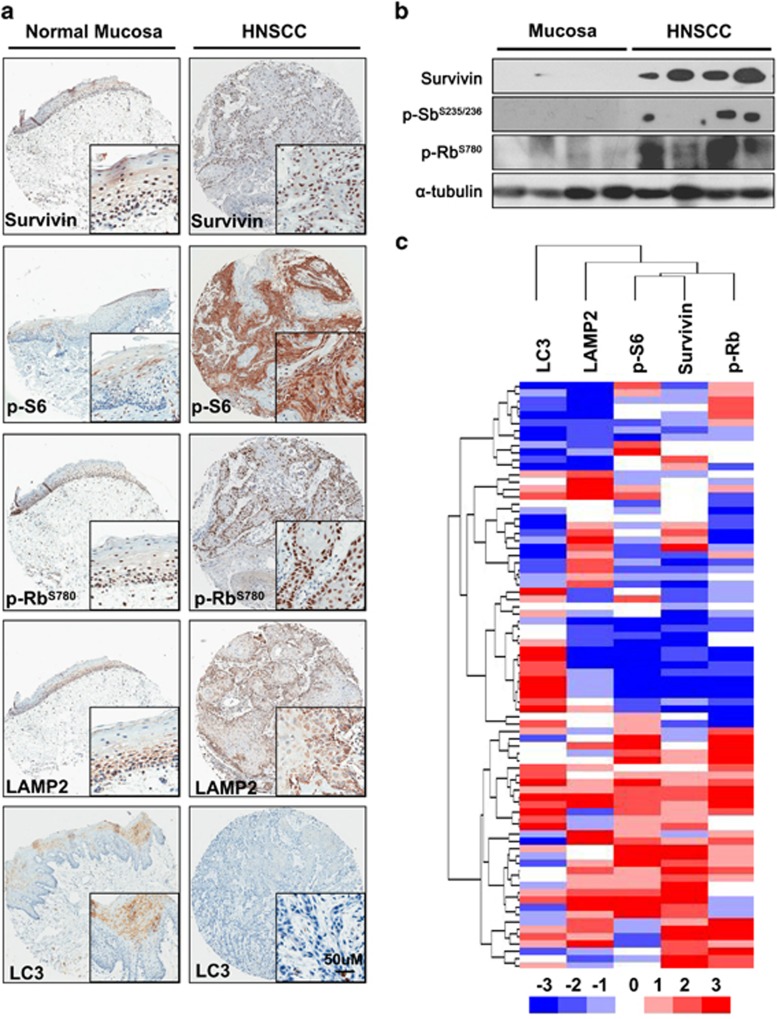
High expression of survivin in human HNSCC. (**a**) High expression of survivin, p-Rb, p-S6 and low expression of LC3 in human HNSCC tissues as compared with normal mucosa. (**b**) High expression of survivin correlated with pS6 and pRb were detected in human HNSCC tissues as compared with normal mucosa. (**c**) Hierachal clustering shows correlation of survivin with p-Rb, p-S6,LAMP2 and LC3

To further investigate the protein expression and correlation of survivin in human tissues, we conducted immunohistochemical analyses on survivin and other important kinases and prosurvival molecules, including p-Rb and p-S6, as well as autophagy related proteins, such as LAMP2 and LC3, through serial human HNSCC tissue arrays (*n*=43). The results were then compared with those in normal head and neck mucosa samples (*n*=10). The representative nuclear immunohistochemical staining results of survivin and p-Rb, the cytoplasm immunostaining of p-S6, LAMP2 and LC3 were shown in Figure 6a. The results revealed that survivin was ubiquitously expressed in tumor tissues. And the strong stainings of p-S6, p-Rb and LAMP2 were also observed in HNSCC tissues. Meanwhile, low expression levels of survivin, p-Rb and LAMP2 were principally detected in the basal layer of the oral mucosa. LC3 was mostly expressed in the oral mucosa but was significantly reduced in HNSCC tissues. Moreover, western blot analysis confirmed that the higher survivin level in human HNSCC tissues (*n*=4) than in normal oral mucosa, which was positively correlated with p-S6 and p-Rb levels (Figure 6b). Hierarchical clustering and linear correlation showed the close correlation among survivin, p-Rb, p-S6 and the lysosome membrane protein LAMP2 in HNSCC. These data suggested that the high expression level of survivin in HNSCC might be closely correlated with cell proliferation, mTOR pathway and autophagy (Figure 6c).

## Discussion

The persistent overexpression and activation of the survivin pathway in numerous cancers, including HNSCC, have indicated that survivin is a preferential therapeutic target for cancer treatment. This fact has been supported by developing preclinical and clinical investigations.^26, 27, 28^ However, the detailed therapeutic efficacy and underlying mechanisms in HNSCC still need to be addressed. In the present study, we corroborated that survivin overexpression in human HNSCC cell lines and tissues, which was correlated with cell cycle regulators, autophagic genes, and mTOR pathway, as indicated by the compelling correlations of p-S6, p-Rb, Cyclin D1, LC3 and LAMP2 in human HNSCC. Survivin overexpression is remarkably related to the suppression of autophagy in HNSCC as compared with the normal oral mucosa. The therapeutic efficacy of survivin inhibition was evaluated by administering its selective suppressant YM155 in xenograft and transgenic mice models of HNSCC. Results showed that survivin inhibition reduced tumor growth and induced apoptosis and autophagy. And autophagy induced by YM155 was confirmed to be another pattern of programmed cell death in HNSCC mice models and cell lines. Moreover, survivin inhibition by YM155 significantly increased the antitumor effect of docetaxel without extra toxicity to the mice.

Survivin is an attractive potential target for cancer therapies because it is rarely expressed in normal cells but remarkably unregulated in several types of cancers.^29^ The results of HNSCC tissue array that included 57 cases have also confirmed the higher expression of survivin in the nuclei of cancer cells than in those of normal mucosa. Survivin is a nodal protein that controlled several prosurvival signaling pathways and is involved in both apoptosis and autophagy. The increased survivin in cancer cells after chemotherapy has been verified and responsible for the resistance against multiple types of antitumor therapies. Therefore, survivin inhibition is selected alone or in combination with other antitumor drugs as a potential strategy for curing cancer. YM155 reduces the expression of survivin at the transcriptional level by moderating the 2 Kb promoter region of the survivin gene, and induces the dissociation of the paraspeckle 54 kDa regulatory nuclear RNA-binding protein (p54nrb) from interleukin enhancer-binding factor 3 (ILF3), which results in the different subcellular localizations of ILF3 and p54nrb and eventually in survivin downregulation.^13^ Therefore, YM155 is selected for the present study to explore the therapeutic efficacy of targeting survivin in HNSCC.

YM155 significantly induces apoptosis and autophagy *in vitro* and *in vivo* with survivin downregulation, thereby proving the efficacy of YM155 in HNSCC treatment. Autophagy has been considered to be simultaneously induced by antitumor therapies and is evaluated as a prosurvival strategy for preventing cell death. Activated autophagy in response to stress can enable long-term survival when apoptosis is defective.^30, 31^ However, although autophagy can promote tumor cell survival under metabolic stress, various tumors may paradoxically suppress autophagy.^32^ Moreover, the induction of autophagic cell death has been proposed as a possible tumor suppression mechanism.^33, 34, 35^ To determine the exact role of autophagy in the present study, CQ, a widely used inhibitor of autophagy flux, was performed in combination with YM155. Results showed that CQ combined with YM155 significantly suppressed the conversion of LC3-I to LC3-II, and reduced expression of cleaved PPARP, indicating that suppressed autophagy enhanced the YM155-induced cell death. Apoptosis and autophagy are closely related because of numerous shared proteins, including survivin and Beclin1. The direct interactions between survivin and Beclin1 regulate the TRAIL-induced apoptosis by controlling the degradation of survivin in a ubiquitin proteasome-dependent manner, reflecting the functional relationship between autophagy and apoptosis.^24^ In the present study, we demonstrated that YM155-induced autophagy was dependent on Beclin1 upregulation probably because of the destruction of the interactions between survivin and Beclin1 partially. These disrupted interactions caused the release of the functional Beclin1 and further promoted autophagy, reflecting another possible mechanism of the interactions between apoptosis and autophagy.

The Akt/mTOR signaling pathway is the main upstream regulation pathway of survivin expression in different types of cancer.^36^ This finding coincides with our previous findings, in which we have proven that the HNSCC in *Tgfbr1/Pten* 2cKO mice was related to survivin expression through mTOR activation. Survivin inhibition by YM155 downregulates the activation of the Akt/mTOR/S6 signaling pathway in a dose-dependent manner, which may delay tumor onset; this result is similar to the mTOR suppression in the transgenic mouse model of HNSCC in our previous report.^25^ These findings prove the essential role of survivin in carcinogenesis and further progression of HNSCC. Considering the similarities between the inhibition of survivin and mTOR in our HNSCC model and the extensively reported effects of mTOR inhibitors on cancer stem cells,^37, 38^ future studies should explore the combined therapeutic effect of YM155 on cancer stem cells. And interestingly, a recent study on pluripotent stem cell-derived teratoma has suggested that YM155 promoted the differentiation of tissues consisting of iPSC by selectively eliminating the undifferentiated pluripotent cells.^39^ Based on this study and our present research, we speculated that YM155 exhibits a significant potential for cancer therapy because of its possible role in the selective destruction of dedifferentiated cancer cells or undifferentiated cancer stem cells. Based on above, we predicate that survivin inhibitor may have synergic effects with traditional chemoreagents, such as platiums and taxanes, in HNSCC, which have been proven by previous studies and preclinical researches in various cancers.^15, 40, 41, 42^ Therefore, we examined the synergistic effects of YM155 combined with docetaxel in HNSCC using xenograft models. Results showed that YM155 combined with docetaxel exhibited better antitumor effects than either chemoreagent alone without additional toxicity, showing synergistic effects on HNSCC. Further preclinical investigations on YM155 as a cancer stem cell-targeted remedy alone or in combination with traditional chemoreagents should be conducted.

In summary, the overexpression of survivin in HNSCC tissues represents the most important factor that predicts poor prognosis and resistance to chemotherapy and/or radiotherapy. The clinical application of survivin as a molecular target in HNSCC therapy significantly benefits HNSCC treatment alone or in combination with traditional cancer therapies. The present study may provide rational strategies for the future clinical evaluation of YM155 in an adjuvant setting as part of a targeted molecular strategy after definitive treatment.

## Materials and methods

### Chemicals and reagents

All reagents were purchased from Sigma-Aldrich (St Louis, MO, USA), unless indicated. Primary antibodies for p-mTOR (Ser2448), mTOR, p-S6 (Ser235/236), S6, p-Rb(Ser780), Atg5, LC3, Bcl2l1, Bcl2, Beclin-1 and cleaved-poly (ADP-ribose) polymerase (PARP) were purchased from Cell Signaling Technology (Danvers, MA, USA). Primary antibody of LAMP2 were purchase from Proteintech (Chicago, IL, USA), Primary antibody of SQSTM1/p62 were purchase from Novus Biological (Littleton, CO, USA). Cyclin D1 antibody was purchased from Santa Cruz (Santa Cruz, CA, USA). Sepantronium bromide (YM155) was purchased from Selleck (Houston, TX, USA).

### Cell culture

Human head neck cancer cell lines SCC4, SCC9, SCC15, SCC25, and CAL27 were purchased from the American Type Culture Collection (ATCC; Manassas, VA, USA). Cell lines HSC3, KCCT-873 and OSC19 were kindly provided by Dr. Raji Puri from FDA, USA to ABK. Human oral keratinocytes (HOK) (ScienCell Research Laboratories, Carlsbad, CA, USA) was used as normal control. All cell lines were cultured in DMEM containing 10% fetal bovine serum at 5% CO_2_ and 37 °C in a humidified incubator as previous described.^25, 43, 44, 45, 46^ YM155 was dissolved in DMEM to produce a stock solution that was aliquot and stored at −20 °C. Chloroquine (CQ; Sigma) was dissolved in DMEM to produce a 50 mmol/l stock solution that was aliquot and frozen at −20 °C.

### Cell immunofluorescence and confocal microscopy

Immunofluorescence was performed as previous described.^36^ Cells immunofluorescence were photographed by confocal laser scanning microscopy (CLSM-310, Zeiss, Jena, Germany). For immunofluorescent staining, the cells were fixed with 4% paraphamydehde after indicated treatment. After incubation with primary antibody, the slides were incubated with fluorophore-conjugated secondary antibody with 4′, 6′-diamidino-2-phenylidole (DAPI) (Jackson ImmunoResearch Laboratories, Inc, West Grove, PA, USA) for 1 h in the dark at room temperature. The primary antibodies included the following: Suvivin, LC3, p-S6^S235/236^ antibodies (Cell Signaling Technology, 1 : 200). Sodium borohydride and Sudan Black B (Sigma, St Louis, MO, USA) were used to reduce aldehyde and lipofuscin-induced fluorescence. Confocal microscopy images were obtained using a Zeiss LSM 510 NLO META confocal microscope (Zeiss).

### Deoxynucleotidyl transferase-mediated dUTP nick end labeling (TUNEL) assay

Apoptotic cells in tumor tissues were measured by the terminal deoxynucleotidyl transferase-mediated dUTP nick end labeling (TUNEL) assay as previous described.^16^ TUNEL was performed using the in situ cell death detection kit, POD (Roche, Mannheim, Germany) according to the manufacturer's instructions. Then six representative high power field areas of each section of vehicle treated (*n*=5) and YM155-treated group (*n*=5) without necrosis were selected. Both apoptotic cells and total cells were counted under microscope.

### Western blots and immunoprecipitation

After indicated treatment, cells were lysed, and their total protein was separated using 12% SDS-PAGE electrophoresis and transferred onto PVDF membranes (Millipore Corporation, Billerica, MA, USA). Then blots were developed by Enhanced chemiluminescence detection kit (West Pico, Thermo, Hudson, NH, USA). GAPDH on the same membrane was used as a loading control.^25, 43, 44, 45, 46^ For immunoprecipitation, CAL27 cells were harvested after treatment with YM155. After centrifugation, the supernatant containing proteins were collected and incubated with survivin antibody pre-coated Dynabeads (Invitrogen, CA, USA) for 30 min. Beads were washed and collected with magnetic stand, and then mixed with Loading buffer for further western blots analysis.

### Establishment and YM155 treatment of CAL27 subcutanous xenograft model

All animal studies include nude mice and transgenic mice were approved and supervised by Animal Care and Use Committee of Wuhan University and conducted in accordance with the NIH guidelines for the Care and Use of Laboratory Animals. Female athymic nude mice (18–20 g; 6–8weeks of age) were obtained from the Experimental Animal Center of Wuhan University in pressurized ventilated cage according to institutional regulations. Mice were housed in appropriate sterile filter-capped cages in Experimental Animal Center of Wuhan University and fed and watered *ad libitum*.

For xenografts, the mice were injected subcutaneously CAL27 cells (4 × 10^6^ in 0.2 ml of serum-free medium) in the flank when cells were exponentially growing. After the tumors were established, the mice were divided into two groups randomly, which received YM155 (10 mg/kg i.p. twice per week; *n*=5) or normal saline (vehicle, 100ul i.p. 2/week; *n*=5) infusion for 3 weeks for xenograft and 2 weeks for orthotopic tongue tumors, respectively. Tumor growth was determined by measuring the size of the tumors 3 times per week for xenograft and 2 times per week for orthotopic tongue tumors. The formula (width^2^ × length)/2 was used to calculate tumor volumes. The mice were euthanized at the indicated time points and the tumors were harvested for the following immunohistochemical analysis and western blotting analysis.

For combined therapy of YM155 with docetaxel. After tumors were established, the mice were divided into four groups randomly, which received normal saline (vehicle, 100 ul i.p. 2/week; *n*=5), YM155 (10 mg/kg i.p. twice per week; *n*=5), docetaxel 20 mg/kg per week and combined docetaxel and YM155 infusion. The mice were euthanized at the indicated time points and the tumors were harvested for successively histology and molecular analysis.

### Chemopreventive study on *Tgfbr1/Pten* combined conditional knock out (2 cKO) mice

The *Tgfbr1/Pten* 2cKO mice (*K14-Cre*^*ERtam*^; *Tgfbr1*^flox/flox^; *Pten*^flox/flox^) were maintained as previously described.^47, 48^ The *Tgfbr1/Pten* 2cKO mice and their controls (*Tgfbr1*^*flox/flox*^*; Pten*^*flox/flox*^) were from the same litter with mixed genetic background of C57BL/6; FVBN; CD1; 129. The tamoxifen application procedure has been previously described.^47, 48^ Only 4- to 8-week-old male and female *Tgfbr1/Pten* 2cKO mice were included in this study. For in chemopreventive assay, 2 weeks after the last dose of oral tamoxifen application of the *Tgfbr1/Pten* 2cKO mice were randomized into a control group (100 ul PBS. i.p. *n*=5 mice) or a group that received YM155 (10 mg/kg i.p. twice per week, *n*=6 mice), based on our pilot study on the tumorigenesis and survival of 2cKO mice. At the end of studies, mice were euthanized using CO_2_, tissues were harvest for histology and Western blot analysis.

### Human HNSCC tissues array, immunohistochemistry, scoring system, hierarchical clustering and data visualization

HN803 tissue arrays which contain 10 cases of normal tongue mucosa, four cases of lymph node metastasis and 57 confirmed cases of HNSCC were obtained from Biomax US (Rockville, MD, USA). The tissue array clinical data, including pathological classification and TNM classification were also provided by Biomax US. Thirty pathologically confirmed human SCC specimens, with corresponding pericancerous normal salivary gland tissues were collected at the Hospital of Stomatology, Wuhan University. The procedures were performed in accordance with the guidelines of National Institutes of Health regarding the use of human tissues and approved by the institute review board of the Ethics Committee of the Hospital of Stomatology, Wuhan University. Immunohistochemistry were performed as previous described by incubated with survivin, LAMP2, p-S6^S235/236^ (Cell Signaling Technology, 1 : 200), or LC3, pRb^Ser780^ (Abcam, Cambridge, UK, 1 : 200) antibody overnight at 4 °C. The antibody binding was detected by horseradish peroxidase-conjugated secondary antibody using a diaminobenzidine substrate kit (Dako, Carpinteria, CA, USA) according to the manufacturer's protocol as previous described.^25, 43, 44, 45, 46^ The negative control slides were obtained by using PBS buffer instead of the primary antibody. All slices were scanned using an Aperio ScanScope CS scanner (Epistem, Cambridge, MA, USA) and quantified using Aperio Quantification software (Version 9.1, Epistem) for membrane, nuclear, or pixel quantification. Four random areas were selected for scanning and quantification. Histoscore of membrane and nuclear staining was calculated as a percentage of different positive cells using the formula (3+) × 3+(2+) × 2+(1+) × 1. Histoscore of pixel quantification was calculated as total intensity/total cell number. The threshold for scanning of different positive cells was set according to the standard controls provided by Aperio. And the sections of *gfbr1/Pten* 2cKO tongue SCC samples were analyzed using immunohistochemistry by incubating antibodies against Survivin, LC3, LAMP2, p-Akt^S473^, p-S6^S235/236^ (CST, 1 : 200), SQSTM1, Bcl2 (Abcam, 1 : 200), Ki-67 (Santa Cruz, 1 : 400).

The hierarchical analysis was performed using Cluster 3.0 with average linkage based on Pearson's correlation coefficient as the selection variable on the unsupervised approach. The staining scores from immunohistochemistry and TUNEL assay were converted into scaled values centered on zero in Microsoft excel (Microsoft, Seattle, CA, USA), and the results were visualized using the Java TreeView 1.0.5 (http://jtreeview.sourceforge.net/). The data were arranged according to their relationships.

### Antibody array and determine of apoptosis

Apoptosis induced by YM155 was determined by (a) MTT assays, (b) typan blue exclusion (TBE) assays, (c) cytochrome c release assays, (d) caspase-9 activity measurements, (e) Annexin V-FITC/PI double staning assays, (f) apoptosis antibody array detections, (g) mitochondrial membrane potential changes and (h) quantitation of cytoplasmic histone-associated DNA fragments with Cell Death Detection ELISA^PLUS^ kit (Roche; Applied Science, Berlin, Germany) and (i) western blots analysis for PARP cleavage.^25, 43, 44, 45, 46^

### Detection and monitor of autophagy

Induction of autophagy by YM155 in CAL27 cells was determined according to guideline of autophagy as followed: (a) LC3 dot punctae detection by LC3 immunofluroscence as previously described. Cells with more than 5 bright LC3 dot punctae in the cytoplasm surrounding the nuclear were consider as a LC3-positive cells. LC3 dot punctae were quantified according to the guideline in detecting autophagy by counting percentage of LC3-positive cell; (b) western blot analysis for LC3II/LC3I ratio and expression of SQSTM1/p62, LAMP2.

### Statistical analysis

Data analyses were conducted using Graph Pad Prism version 5.00 for Windows (Graph-Pad Software Inc, La Jolla, CA, USA).^25, 43, 44^ One-way ANOVA followed by the post-Dunnett or Bonferroni multiple comparison tests were used to analyze the differences in protein levels among each group. The Mann–Whitney *U*-test and student *t*-test was used to evaluate differences in the protein expression, immunohistochemistry and total tumor area of the mice YM155-treated and vehicle-only group. Log rank statistics were used to evaluate survival in the YM155 and control groups. Mean values±S.E.M. with a difference of *P*<0.05 were considered statistically significant.

## Abbreviations

Akt: protein kinase B, PKB
Bax: bcl-2-like protein 4
Bad: the Bcl-2-associated death promoter
cIAP-1: cellular inhibitor of apoptosis 1
CL-caspase3: cleaved caspase3
CL-PARP: cleaved poly (ADP-ribose) polymerase
DAPI: 4′,6-diamidino-2-phenylindole
Diablo: direct IAP binding protein with low pI
FADD, DR4/5: death receptor 4/5 fas-associated protein with death domain
HIF-1α: Hypoxia-inducible factor 1α
HNSCC: head neck squamous cell cancer
HO-2: Heme oxygenase -2
HTRA2, HSP27/70: heat shock protein 27/70 HtrA serine peptidase 2
LAMP2: lysosome-associated membrane protein 2
LC3: microtubule-associated protein 1 light chain 3B
mTOR: mammalian Target Of Rapamycin
MTT: 3-(4,5-dimethyl-2-thiazolyl)-2,5-diphenyl-2-H-tetrazolium bromide
Rb: retinoblastoma gene
SQSTM1: Sequestosome 1, p62
Tam: tamoxifen
TUNEL: Terminal deoxynucleotidyl transferase dUTP nick end labeling
YM155: sepantronium bromide
