# Supplementary material for: Dual induction of apoptotic and autophagic cell death by targeting survivin in head neck squamous cell carcinoma
Source: Cell Death Dis. 2015 May 28;6(5):e1771–. doi: 10.1038/cddis.2015.139 (PMC4669714; doi:10.1038/cddis.2015.139)
Supplement: Supplementary Information [file cddis2015139x1.doc]

**Supplementary Figure Legends**

**Supplementary Figure 1** YM155 induces apoptosis in HNSCC cell lines. (a) The protein expression of Survivin and related kinases in various HNSCC cell lines. (b) The cell growth of HSC3 cell line after YM155 treatment. (c) YM155 suppress the protein and mRNA expression of Survivin in CAL27 cells. (d) Quantification analysis of apoptosis relative index of human apoptosis antibody arrays. (e) ELISA assay of cytochrome C release by YM155 treatment. (f) Relative Caspase 9 activity of CAL27 and HSC3 cells by YM155 treatment.

**Supplementary Figure 2** YM155 induces autophagy in human HNSCC cell lines. (a) Western blot analysis showed YM155 increased LC3II/LC3I ratio, LAMP2 and Beclin1 expression, reduced SQSTM1 expression in HSC3 cells. (b) Western blot analysis showed YM155 increased LC3II/LC3I ratio in CAL 27, and reduced LAMP2 after 1.5 h.

**Supplementary Figure 3** YM155 induces cell death dependent on Beclin1. (a) Immunoprecipitation shows that the Beclin1 binded with Surivivin as well as Survivin was downregulated after YM155 treatment. (b) Representative dot plot of flow cytometry showed cell death induced by YM155 but siRNA against survivin was dependent on Beclin1 in CAL27 cells.

**Supplementary Figure 4** YM155 treatment reduces CAL27 heterotopic xenograft tumor. (a) Shows schematic treatment strategy of tumor-bearing mice in vehicle and treatment group. The red arrow depicts the initating time of injection. (b) Representative picture shows tumor growth condition in YM155 group and vehicle group at early-stage and late-stage of treatment. (c) Representative 28 days tumor xenograft sample treated with vehicle and YM155. (d) Tumor growth curve of YM155 group versus vehicle group. (e) Tumor burden were assessed in YM155 and control group at day 28; Mean±SEM; **, *P*＜0.01; student *t* analysis.

**Supplementary Figure 5** YM155 increases anti-tumor effect of docetaxel in CAL27 cell line and xenograft. (a) Western blot analysis showed docetaxel treatment increased expression of survivin in CAL27 cells, while concomitant treatment by YM155 attenuated the increase survivin by docetaxel. (b) Immunofluorescence showed nuclear expression of survivin increased by docetaxel treatment but attenuated by concomitant YM155 treatment. (c) Tumor burden of YM155, docetaxel and combined chemotherapeutics of CAL27 xenograft. ***, *P*＜0.001 as compared with vehicle only group. ^###^, *P*＜0.001 as compared with combined treatment only group. (d) Mice weight of YM155, docetaxel and combined chemotherapeutics of CAL27 xenograft. **, *P*＜0.001 as compared with vehicle only group.
