# Supplementary figures and images for: Dual induction of apoptotic and autophagic cell death by targeting survivin in head neck squamous cell carcinoma
Source: Cell Death Dis. 2015 May 28;6(5):e1771–. doi: 10.1038/cddis.2015.139 (PMC4669714; doi:10.1038/cddis.2015.139)

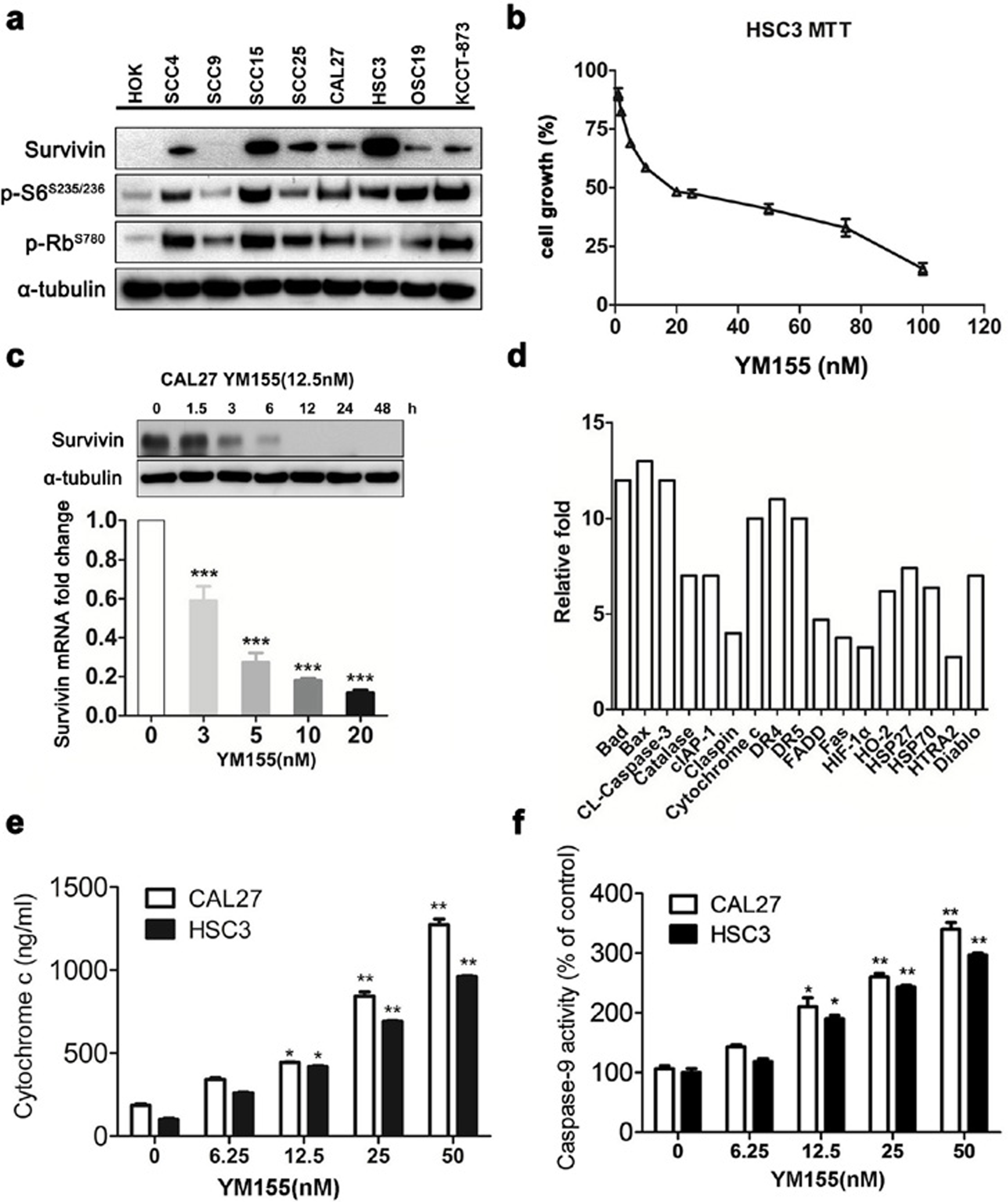

Supplement: Supplementary Figure 1 [file cddis2015139x2.tif]

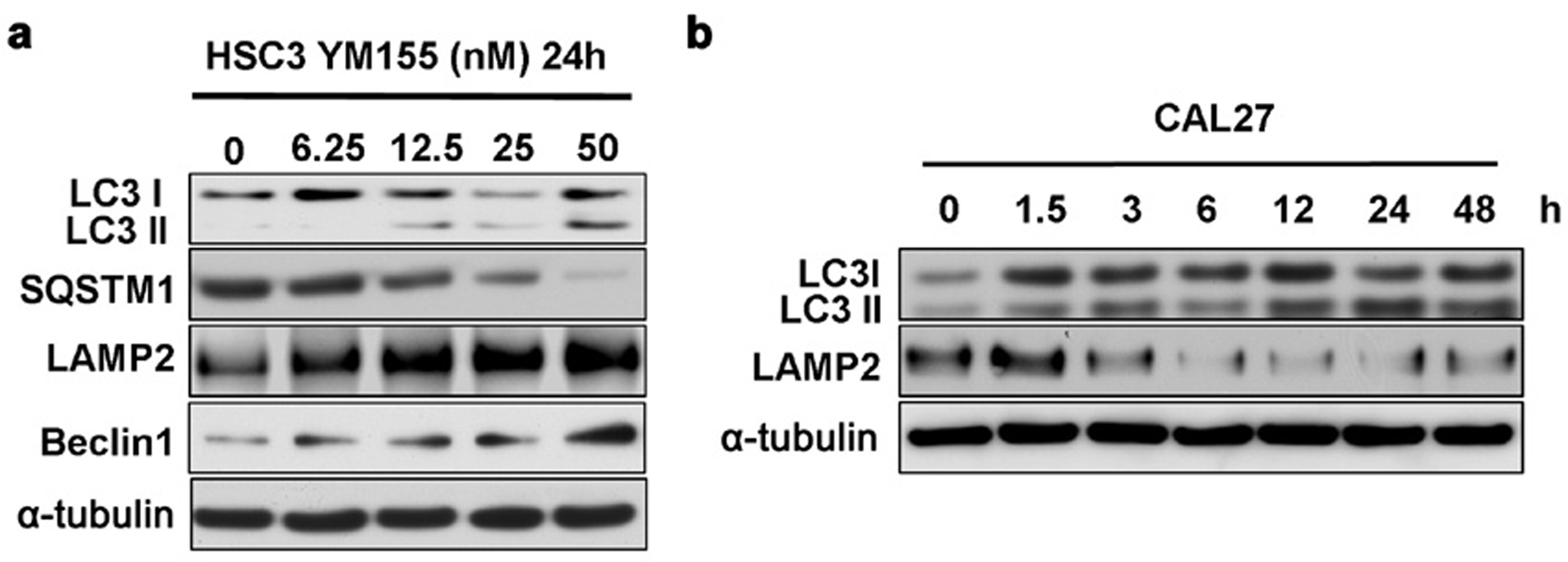

Supplement: Supplementary Figure 2 [file cddis2015139x3.tif]

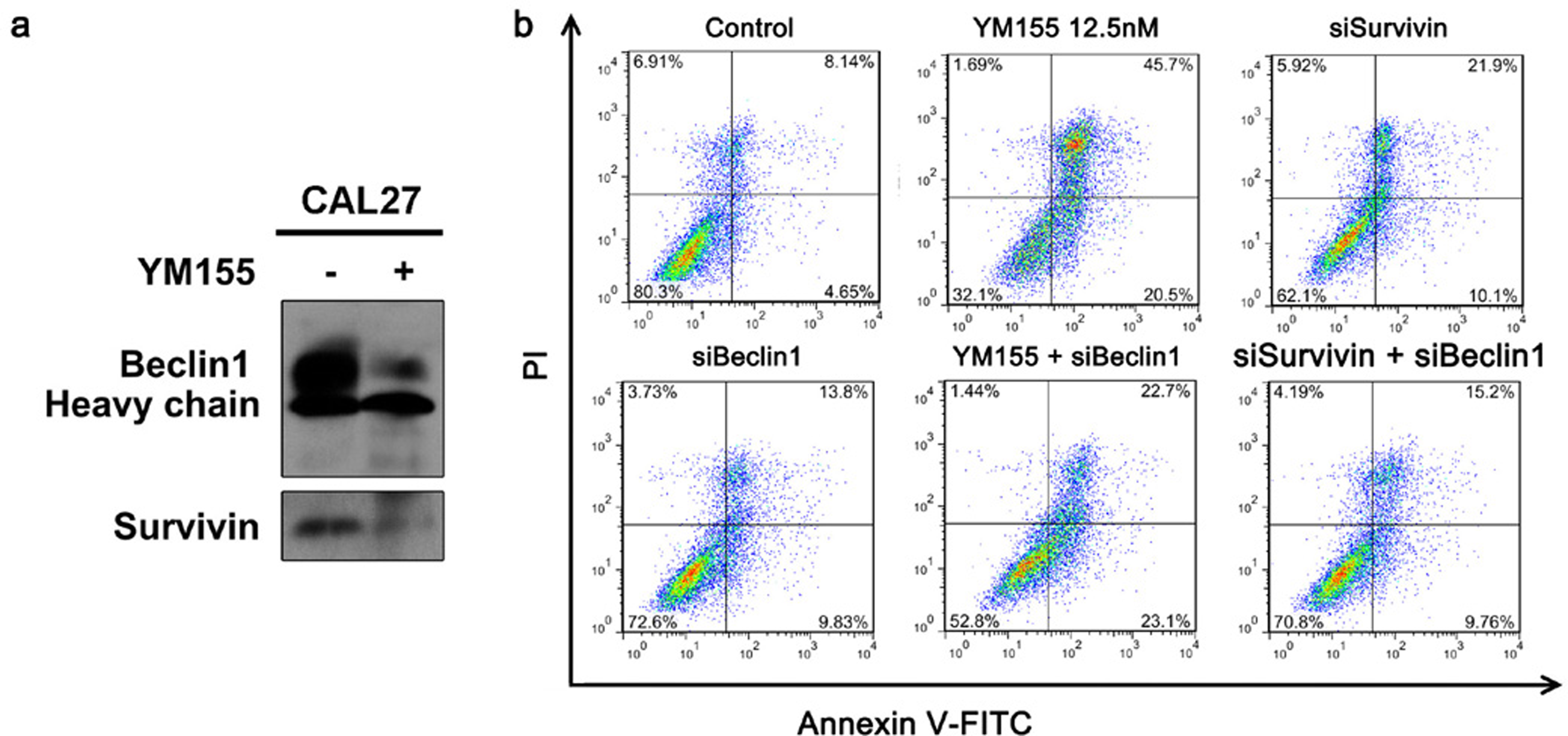

Supplement: Supplementary Figure 3 [file cddis2015139x4.tif]

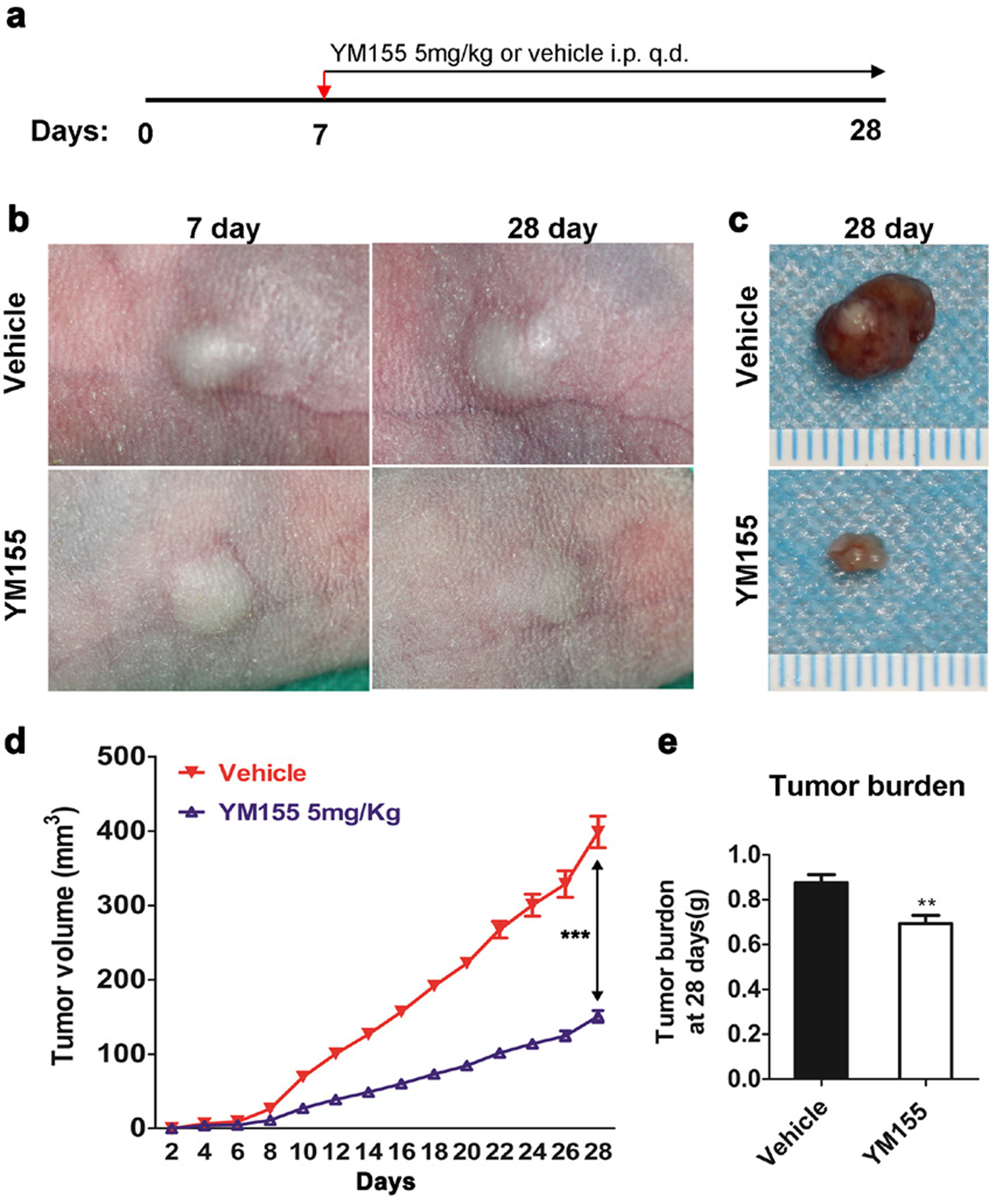

Supplement: Supplementary Figure 4 [file cddis2015139x5.tif]

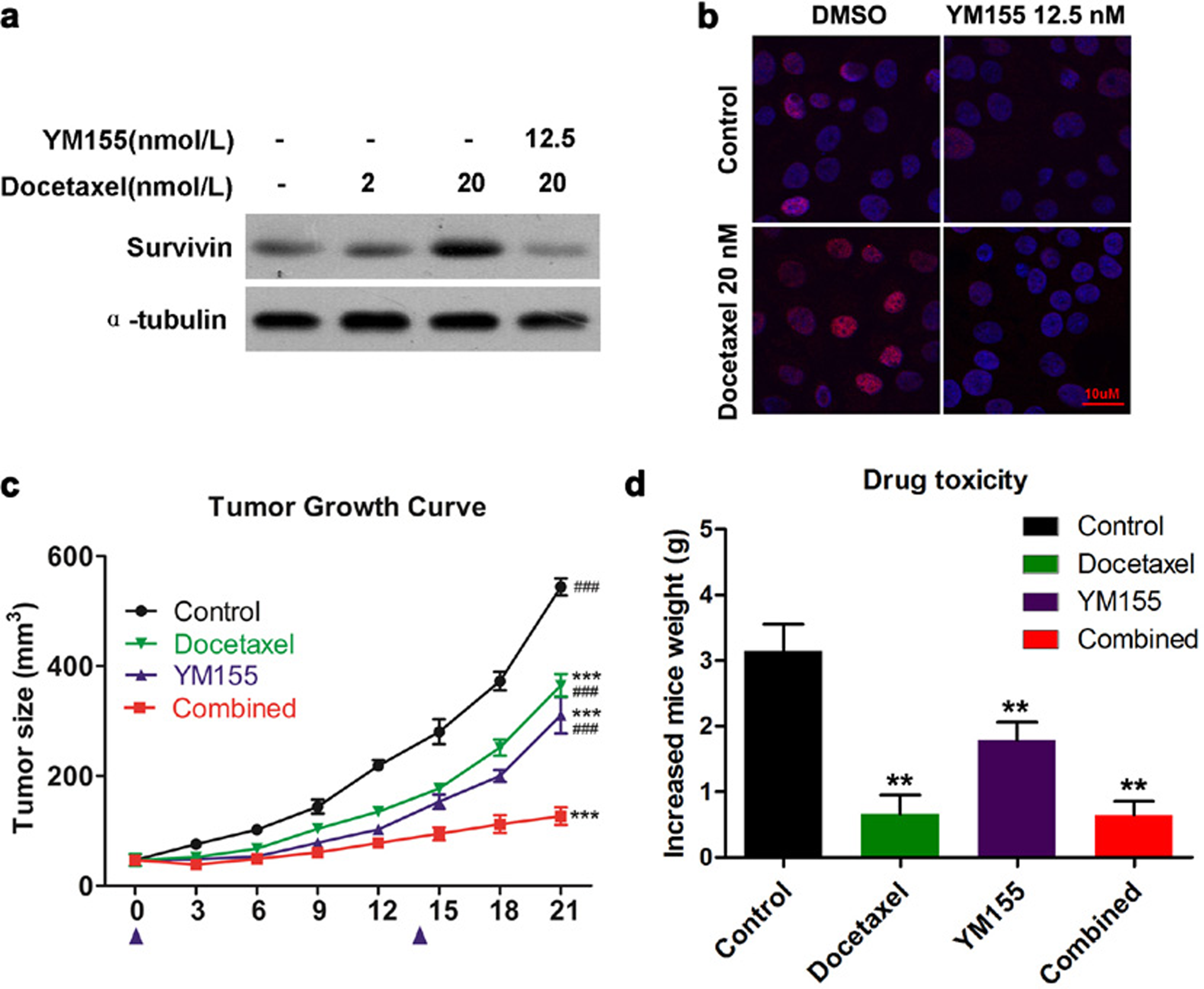

Supplement: Supplementary Figure 5 [file cddis2015139x6.tif]
